# Effect of Stoichiometry on Shape Memory Properties and Functional Stability of Ti–Ni–Pd Alloys

**DOI:** 10.3390/ma12050798

**Published:** 2019-03-08

**Authors:** Yuki Hattori, Takahiro Taguchi, Hee Young Kim, Shuichi Miyazaki

**Affiliations:** 1 Graduate School of Pure and Applied Sciences, University of Tsukuba, Tsukuba, Ibaraki 305-8573, Japan; y.hattori2017@gmail.com (Y.H.); tagutaka43@gmail.com (T.T.); 2 Faculty of Pure and Applied Sciences, University of Tsukuba, Tsukuba, Ibaraki 305-8573, Japan; 3 Foundation for Advancement of International Science, Tsukuba, Ibaraki 305-0821, Japan

**Keywords:** shape memory effect, martensitic transformation, high-temperature shape memory alloy, thermal cycling, precipitation, stoichiometry, differential scanning calorimetry

## Abstract

Ti–Ni–Pd shape memory alloys are promising candidates for high-temperature actuators operating at above 373 K. One of the key issues in developing high-temperature shape memory alloys is the degradation of shape memory properties and dimensional stabilities because plastic deformation becomes more pronounced at higher working temperature ranges. In this study, the effect of the Ti:(Ni + Pd) atomic ratio in Ti_x_Ni_70−x_Pd_30_ alloys with Ti content in the range from 49 at.% to 52 at.% on the martensitic transformation temperatures, microstructures and shape memory properties during thermal cycling under constant stresses were investigated. The martensitic transformation temperatures decreased with increasing or decreasing Ti content from the stoichiometric composition. In both Ti-rich and Ti-lean alloys, the transformation temperatures decreased during thermal cycling and the degree of decrease in the transformation temperatures became more pronounced as the composition of the alloy departed from the stoichiometric composition. Ti_2_Pd and P phases were formed during thermal cycling in Ti-rich and Ti-lean alloys, respectively. Both Ti-rich and Ti-lean alloys exhibited superior dimensional stabilities and excellent shape memory properties with higher recovery ratio and larger work output during thermal cycling under constant stresses when compared with the alloys with near-stoichiometric composition.

## 1. Introduction

Shape memory alloys exhibiting reversible martensitic transformation have been considered to be attractive materials for novel actuators due to their greater work output than other smart materials [1,2,3]. Although shape memory alloys have desirable properties for actuators such as high recovery force and large recoverable strain, they have a limitation in their operating temperatures. The maximum operating temperature of commercially available Ti–Ni shape memory alloys is limited to around 350 K due to their low martensitic and reverse transformation temperatures [1,2,3]. In recent years, there have been strong demands for actuators operating at higher temperatures above 373 K (100 °C); therefore, there have been substantial research efforts to develop high-temperature shape memory alloys [4,5,6,7,8,9,10].

It has been well documented that the transformation temperatures of Ti–Ni shape memory alloys can be raised above 373 K by the addition of ternary elements of Zr and Hf in replacement of Ti [11,12,13,14]. The relatively low cost of raw materials makes Ti–Ni–(Zr,Hf) alloys attractive candidates for practical high-temperature shape memory alloys; however, there are still several issues needed to be resolved, including poor workability, large transformation temperature hysteresis and fast degradation of the reversibility of shape memory properties. Therefore, recently there have been considerable efforts to improve the shape memory properties and mechanical properties of Ti–Ni–(Zr,Hf) alloys [15,16,17,18,19,20,21,22,23]. Precipitation hardening has been found to be one of the promising approaches [19,20,21,22,23]. It has been reported that the formation of nanoscale precipitates works very effectively for the improvement of mechanical and thermal cyclic stabilities of the shape memory effect of Ni-rich Ti–Ni–(Zr,Hf) alloys [19,20,21,22,23].

Noble elements, such as Pd, Au and Pt, are also effective in increasing the transformation temperatures of Ti–Ni shape memory alloys when they are added at the expense of Ni. In particular, Ti–Ni–Pd alloys have received significant research attention because of their unique useful properties such as high transformation temperature, small transformation temperature hysteresis, light weight, adequate workability and relatively low price when compared with other high-temperature shape memory alloys such as Ti–Ni–Au and Ti–Ni–Pt [4,13,24,25,26,27]. The addition of Pd reduces the transformation temperature in the range below 10 at.% Pd, then raises the transformation temperature in the alloys with higher Pd contents above 10 at.%. The martensitic transformation start temperature is raised above 373 K by the addition of 25 at.% Pd and increases up to 523 K by the addition of 30 at.% Pd. 

Similar to other high-temperature shape memory alloys, the shape memory properties of Ti–Ni–Pd alloys, particularly in terms of shape recovery ratio and cyclic dimensional stability, become remarkably degraded with increasing the working temperature range. The degradation of shape memory properties is mainly due to fact that plastic deformation becomes more pronounced, and as a result, permanent strain is easily introduced at elevated temperatures. Therefore, one of the key issues in researching Ti–Ni–Pd alloys is the improvement of resistance to permanent deformation upon actuation cycles at higher working temperature ranges. There have been various approaches to improve shape memory properties at higher temperatures such as (1) grain size refinement through severe plastic deformation [28], (2) the addition of quaternary elements [29,30,31] and (3) precipitation strengthening [25,32,33,34,35,36]. The combination of multiple strengthening mechanisms has been found to be effective for increasing strength and improving shape memory properties [37,38]. It has been evident that precipitation strengthening is the most effective method to improve shape memory properties. 

It has been well acknowledged that transformation behavior and shape memory properties of Ti–Ni based alloys are strongly dependent on the ratio of Ti:Ni [13,38,39,40,41,42]. For the Ti–Ni binary alloys, the transformation temperatures decrease considerably with increasing the Ni content in Ni-rich alloys while the transformation temperatures are relatively insensitive to the composition in Ti-rich alloys [13,39]. The shape memory properties can be drastically improved by the formation of Ti_3_Ni_4_ precipitates in Ni-rich Ti–Ni alloys. In Ti–Ni–Pd ternary alloys, the atomic ratio of Ti:(Ni + Pd) is also an important parameter that influences the transformation temperatures [25,42]; however, there have been limited efforts to investigate the effect of the stoichiometry on mechanical and thermal cyclic stabilities of the shape memory effect. In this study, Ti_x_Ni_70−x_Pd_30_ alloys with Ti contents in the range from 49 at.% to 52 at.%, i.e., from Ti-lean to Ti-rich, were fabricated and the effect of the Ti:(Ni + Pd) atomic ratio on the transformation temperatures, microstructures and shape memory properties during thermal cycling under constant stresses were investigated.

## 2. Materials and Methods 

Ti_x_Ni_70−x_Pd_30_ ingots (where x = 49, 49.5, 50, 50.5, 51, 51.5, 52) were fabricated by the arc melting method. Hereafter, the alloys are abbreviated by using their Ti content, i.e. 49Ti, 49.5Ti, 50Ti, 50.5Ti, 51Ti, 51.5Ti and 52Ti. The ingots were melted in an argon atmosphere. The melted ingots were flipped over and re-melted five times to promote homogeneity. The ingots were sealed in a quartz tube under vacuum and homogenized at 1223 K for 7.2 ks. The ingots were sliced into plates 1 mm in thickness using an electro discharge machine (EDM). The plates were cold rolled with intermediate annealing treatment to reach a thickness of 0.17 mm. The final cold rolling ratio was adjusted to 30%. Specimens for X-ray diffraction (XRD), microstructure analysis, differential scanning calorimetry (DSC) measurement and thermal cycling tests under constant stresses were cut from the cold rolled sheets by EDM. These specimens were solution treated at 1173 K for 3.6 ks in Ar-filled quartz tubes and quenched in ice water by breaking the quartz tubes. XRD analysis was carried out at room temperature using a Rigaku Smartlab instrument (Tokyo, Japan) with a Cu Kα source. Microstructure and chemical compositions of phases were investigated by an electron probe microanalyzer (EPMA, JEOL JXA 8530F, Tokyo, Japan) and transmission electron microscopy (TEM, JEOL 2010F). TEM specimens were prepared by twin jet electro-polishing with an electrolyte composed of 85 vol.% methanol (CH_3_OH) and 15 vol.% sulfuric acid (H_2_SO_4_) at 233 K. Transformation temperatures were evaluated from DSC curves. The DSC measurements were carried out using a Shimadzu DSC-60 instrument (Kyoto, Japan) with a heating and cooling rate of 10 K min^−1^. Shape memory properties were investigated by thermal cycling tests under various constant tensile stress levels. The thermal cycling tests were conducted with a heating and cooling rate of 10 K min^−1^ in a nitrogen gas atmosphere. 

## 3. Results and Discussion

### 3.1. Cyclic Responce of Transformation Temperatures 

**Figure 1:**
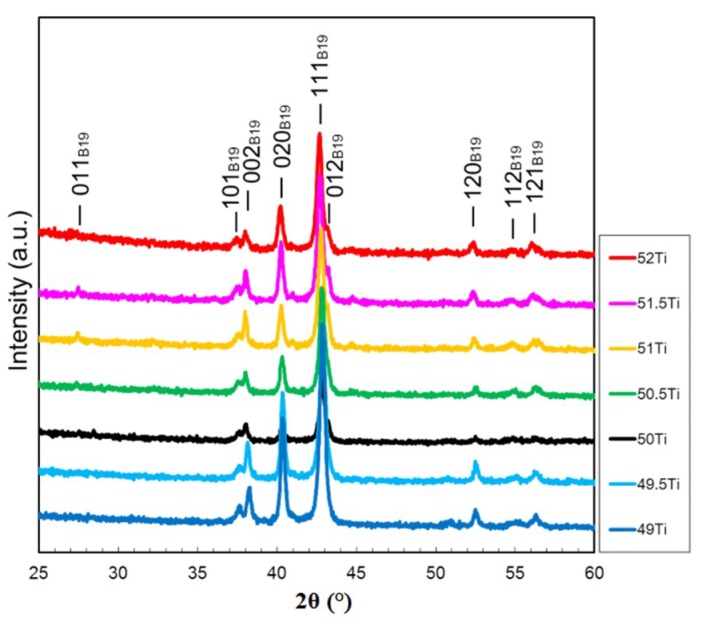
X-ray diffraction (XRD) profiles of the (49–52)Ti alloys solution treated at 1173 K.

Figure 1 shows the XRD profiles obtained at room temperature of the (49–52)Ti alloys solution treated at 1173 K. All the major diffraction peaks were identified as the B19 martensite phase, indicating that the martensitic transformation finish temperatures of all the alloys were higher than room temperature. 

**Figure 2:**
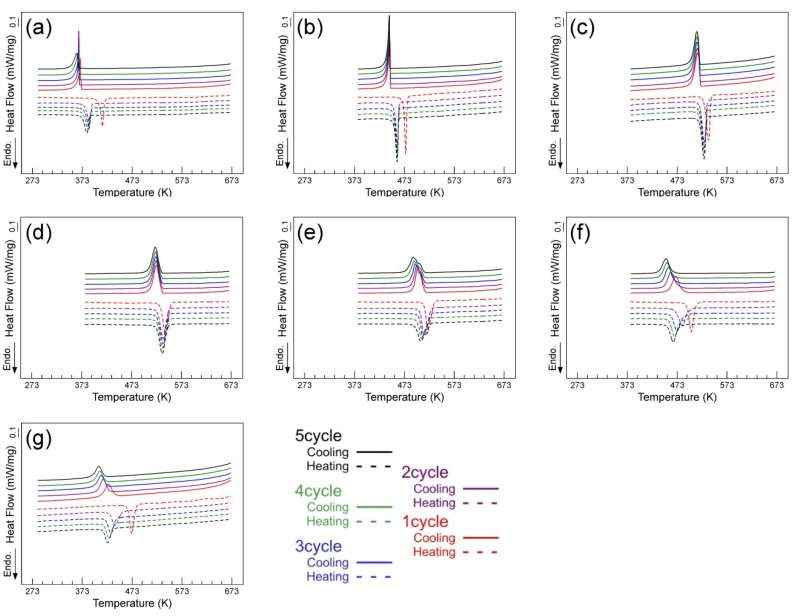
Differential scanning calorimetry (DSC) curves of the (49–51)Ti alloys solution treated at 1173 K. (**a**) 49Ti alloy; (**b**) 49.5Ti alloy; (**c**) 50Ti alloy; (**d**) 50.5Ti alloy; (**e**) 51Ti alloy; (**f**) 51.5Ti alloy; (**g**) 52Ti alloy.

**Figure 3:**
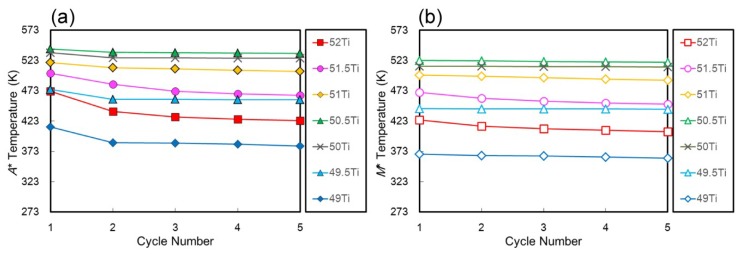
Change of transformation temperatures upon thermal cycling. (**a**) Reverse transformation peak temperature (A*) and (**b**) martensitic transformation peak temperature (M*).

Figure 2 shows DSC curves of five cycles for the (49–52)Ti alloys solution treated at 1173 K. During DSC cycles, the specimens were first heated to a maximum temperature of 673 K, then cooled to 273 K or 373 K depending on the transformation temperature. The results show a pronounced composition dependence of not only the transformation temperature but also the cyclic behavior. The reverse transformation peak temperature (A*) and the martensitic transformation peak temperature (M*) of the (49–52)Ti alloys are plotted as a function of cycle number in Figure 3. In the stoichiometric composition of 50Ti (Figure 2c), A* decreased only slightly from 536 K to 528 K from the first cycle to the second cycle and no noticeable changes were observed in the DSC curves after the second cycle. The DSC curves of the 50Ti alloy show almost a constant value of M* during the thermal cycles. The 50.5Ti alloy (Figure 2d) exhibited similar cyclic behavior as the 50Ti alloy, with the only difference being slightly higher transformation temperatures. With increasing Ti content of the alloy, the cyclic stability of transformation temperatures became worse. Both A* and M* shifted to lower temperatures continuously during repeated cycles in the 51Ti alloy (Figure 2e). In the 51.5Ti (Figure 2f) and 52Ti (Figure 2g) alloys, the decrease in the transformation temperatures, both A* and M*, due to the cycle became more prominent. For a Ti-lean alloy with 49.5Ti (Figure 2b), A* decreased markedly from the first cycle to the second cycle, while it remained almost constant during further successive cycles. The 49Ti alloy (Figure 2a) exhibited a similar decrease in A* from the first cycle to the second cycle, but A* decreased continuously after the second cycle although the decrease was not significant. It is also noted that the change of the transformation temperatures becomes apparent as the composition of the alloy departs from the stoichiometric composition on both Ti-rich and Ti-lean sides. 

**Figure 4:**
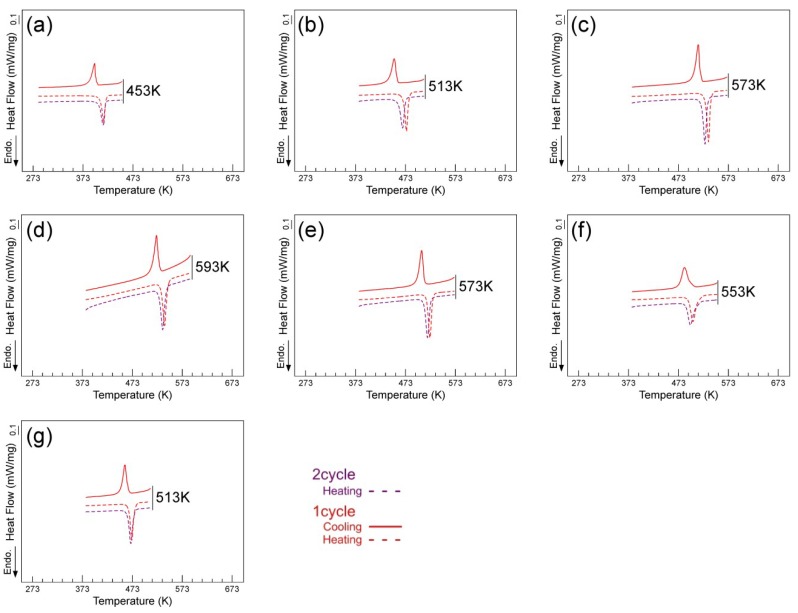
DSC curves of the (49–52)Ti alloys solution treated at 1173 K where the maximum temperature during cycles was adjusted to be about *A*_f_ + 30 K. (**a**) 49Ti alloy; (**b**) 49.5Ti alloy; (**c**) 50Ti alloy; (**d**) 50.5Ti alloy; (**e**) 51Ti alloy; (**f**) 51.5Ti alloy; (**g**) 52Ti alloy.

Figure 4 shows DSC results for the (49–52)Ti alloys where the maximum temperature during cycles was reduced to about *A*_f_ + 30 K in order to evaluate the effect of the upper cycle temperature on the cyclic stability of the transformation temperatures. It is seen that A* of the first cycle is identical in both DSC experiments, making it reasonable to consider that the reverse transformation behavior in the first cycle was not influenced by the maximum temperature. Focusing on the values of A* between the first cycle and the second cycle of the alloys, it is clear that the reduction of the transformation temperature was suppressed with the decrease in the upper cycle temperature. The effect of suppressing the cycle effect is clearer and more prominent in the 49Ti and 52Ti alloys. M* of the first cycle is also found to be influenced by the upper cycle temperature: the higher the upper cycle temperature, the lower the value of M*. Consequently, it is considered that a thermally activated process such as precipitation occurred during thermal cycling.

**Figure 5:**
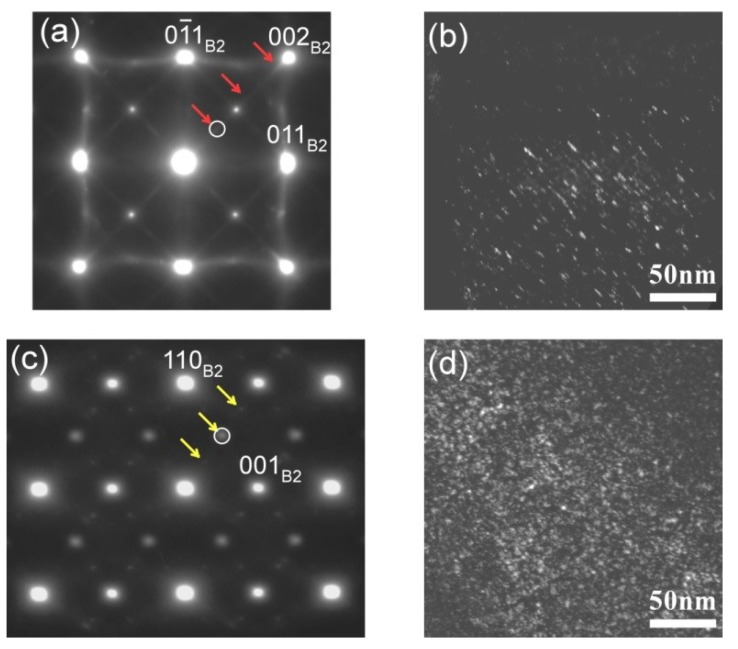
TEM analysis of the specimens cycled five times from 273 K to 673 K. (**a**) SADP of the 52Ti alloy; (**b**) dark field micrograph taken by the diffraction spot labeled by a circle in (**a**); (**c**) SADP of the 49Ti alloy; (**d**) dark field micrograph taken by the diffraction spot labeled by a circle in (**c**).

In order to elucidate the mechanism behind the decrease in the transformation temperatures during thermal cycling, TEM observation was carried out using the samples subjected to DSC measurements. Figure 5a,b shows the selected area diffraction pattern (SADP) with a [001]_B2_ zone axis and a dark field micrograph obtained from the 52Ti alloy cycled five times from 273 K to 673 K. The diffraction pattern reveals extra diffuse streaks noted by arrows in between the primary diffraction spots from the B2 phase. The diffuse streaks were identified as the Ti_2_Pd phase [36,43]. The dark field micrograph taken by using the diffuse streak clearly shows the formation of fine needle-shaped precipitates. Similarly, the selected area diffraction pattern (Figure 5c) and dark field micrograph (Figure 5d) verify the formation of precipitates in the 49Ti alloy after thermal cycling. From the selected area diffraction (Figure 5c), the precipitates were identified to be the P phase [44,45]. The formation of the P phase in the 49Ti alloys is reasonable because the composition of the P phase is reported to be slightly Ti-poor, such as Ti_11_(Ni_7_Pd_6_) [46]. These results imply that the change of the transformation temperatures during thermal cycling in the alloys with off-stoichiometric composition is due to the formation of precipitates. It is also noted that the different types of precipitates are formed in Ti-rich and Ti-lean compositions, i.e., Ti_2_Pd in Ti-rich alloys and P phase in Ti-lean alloys. 

### 3.2. Ti Content Dependence of Transformation Temperature and Microstructure

**Figure 6:**
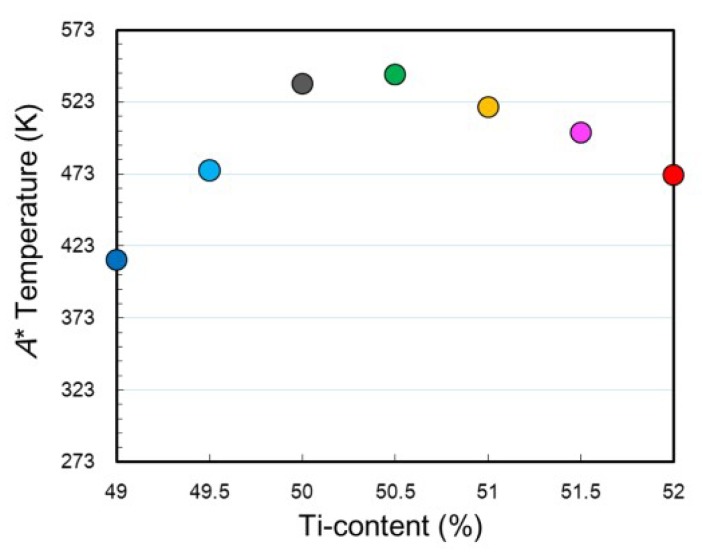
Ti-content dependence of the reverse transformation peak temperature (A*).

In order to evaluate the effect of composition on the transformation temperature which is not affected by thermal cycles, A* of the first cycle is plotted as a function of Ti content in Figure 6. It has been well acknowledged that the transformation temperatures of Ti–Ni binary alloys are insensitive to the Ti content in Ti-rich compositions [13,39,40], which is due to the fact that the composition of the matrix remains unchanged by increasing the volume fraction of the Ti_2_Ni phase with increasing the Ti content. On the other hand, the transformation temperatures of Ti–Ni binary alloys decrease with decreasing the Ti content in the Ti-lean compositions. In contrast, for Ti–Ni–30Pd alloys, A* decreased with decreasing the Ti content from 50 at.% to 49 at.% in the Ti-lean compositions, and it also decreased with increasing the Ti content from 50.5 at.% to 52 at.%. It is expected from the linear extrapolation lines of the both sides that A* takes the peak level at around 50.2 at.% Ti. 

**Figure 7:**
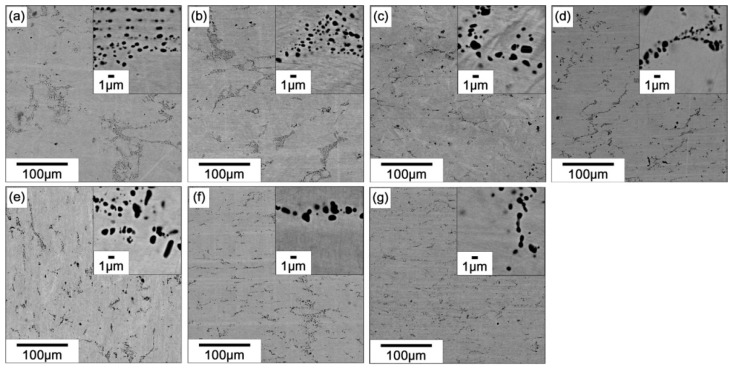
Back-scattered SEM images of the (49–52)Ti alloys solution treated at 1173 K. (**a**) 49Ti alloy; (**b**) 49.5Ti alloy; (**c**) 50Ti alloy; (**d**) 50.5Ti alloy; (**e**) 51Ti alloy; (**f**) 51.5Ti alloy; (**g**) 52Ti alloy.

**Figure 8:**
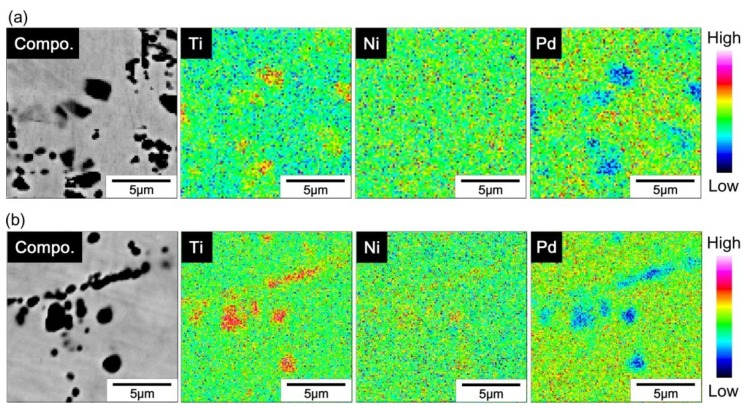
EPMA mapping of elements for (**a**) 49Ti alloy and (**b**) 52Ti alloy.

Figure 7 shows back-scattered SEM images of the (49–52)Ti alloys solution treated at 1173 K. In addition to the matrix phase, i.e., B19 martensite phase, a Ti_2_Ni-type (or Ti_4_Ni_2_O-type) second phase appearing as black particles was observed in all alloys. EPMA results (Figure 8) confirm that the compositions of the second phase particles are similar in both Ti-rich and Ti-lean alloys—the Ti content of the second phase particles is higher than that of the matrix, but the Pd content is lower than that of the matrix. It is important to note that the volume fraction of the second phase particles does not change significantly with the composition of the alloy. It has been reported that the oxygen, which is an unavoidable impurity in Ti–Ni-based alloys, stabilizes the Ti_2_Ni phase [40,47]. 

**Figure 9:**
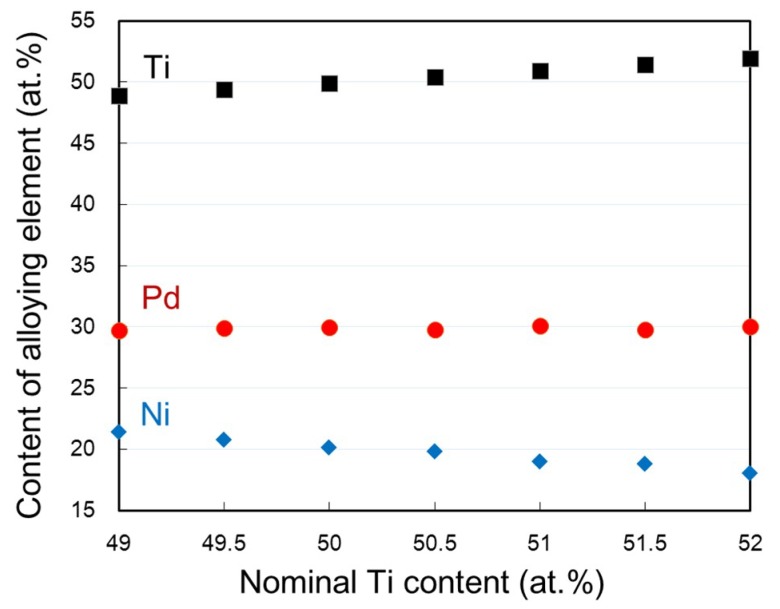
Ti, Ni and Pd contents of the matrix region for the (49–52)Ti alloys measured by EPMA.

Figure 9 shows the Ti, Ni and Pd contents of the matrix region measured by EPMA as a function of the nominal Ti content of the alloy. It is clearly seen that the Ti content of the matrix region increased linearly while the Ni content of the matrix region decreased with increasing the nominal Ti content. The Pd content is not dependent on the nominal Ti content. Also, it is noticed that the measured Ti content of the matrix region was slightly lower than that of the nominal Ti content, which is due to the formation of Ti-rich Ti_2_Ni-type particles. These results indicate that the range of the Ti content in the B2 phase of Ti–Ni–30Pd alloys at 1173 K is wider, particularly in the Ti-rich region, than that of the Ti–Ni binary alloys. It is also evident that the transformation temperature shows the highest value at a composition near the stoichiometric composition (Ti:Ni + Pd = 50:50) and it decreases as the alloy composition deviates from the stoichiometric composition.

### 3.3. Shape Memory Properties

**Figure 10:**
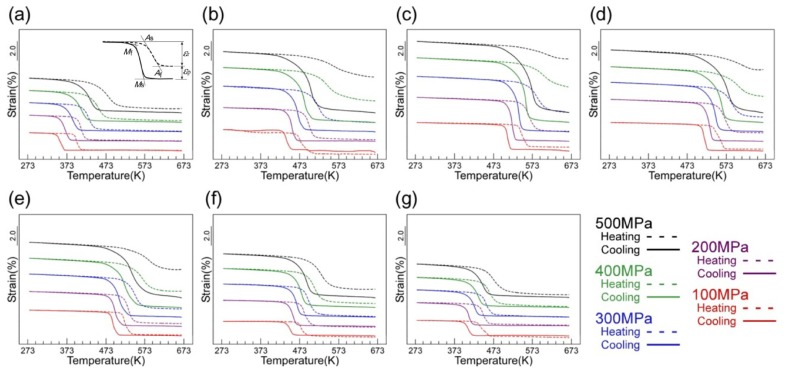
Strain–temperature curves of the (49–52)Ti alloys under various applied stresses. (**a**) 49Ti alloy; (**b**) 49.5Ti alloy; (**c**) 50Ti alloy; (**d**) 50.5Ti alloy; (**e**) 51Ti alloy; (**f**) 51.5Ti alloy; (**g**) 52Ti alloy.

**Figure 11:**
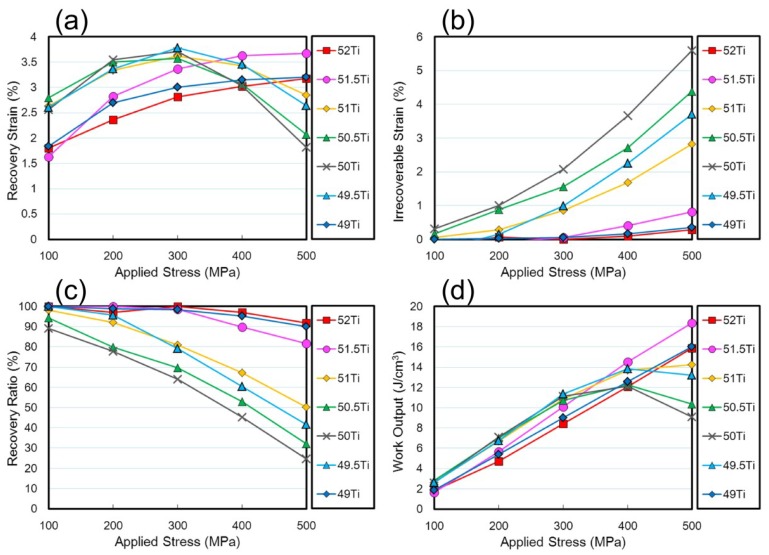
Evaluation of shape memory properties of the (49–52)Ti alloys. (**a**) Recovery strain; (**b**) irrecoverable strain; (**c**) recovery ratio; (**d**) work output.

Thermal cycling tests under various constant stresses were carried out to investigate shape memory properties of the (49–52)Ti alloys. Figure 10 shows the strain–temperature curves obtained during thermal cycling between 273 and 673 K at the stresses of 100–500 MPa. The solid and dashed lines indicate the strain changes upon cooling and heating, respectively. The shape memory effect was confirmed for all alloys—the specimen started to elongate at a certain temperature corresponding to the martensitic transformation start temperature upon cooling and the strain recovered upon heating due to the reverse transformation. Characteristic transformation temperatures, i.e., martensitic transformation start temperature (*M*_s_,) and its finish temperature (*M*_f_,), reverse transformation start temperature (*A*_s_) and its finish temperature (*A*_f_) are shown in Figure 10a as an example. The shape memory properties and dimensional stability were evaluated by measuring the recovery strain (*ε*_r_) and irrecoverable plastic strain (*ε*_p_) and the results are plotted as a function of the applied stress (*σ*_app_) in Figure 11a,b. Also, the shape recovery ratio and work output defined as *ε*_r_/(*ε*_r_ + *ε*_p_) and *σ*_app_ × *ε*_r_, respectively, of the (49–52)Ti alloys are shown in Figure 11c,d. 

It is noticed from Figure 10 and Figure 11 that the dimensional stability of the (49–52)Ti alloys is strongly dependent on the Ti content. In the stoichiometric composition of 50Ti, *ε*_r_ gradually increased with increasing the applied stress until reaching a maximum value of 3.7% at 300 MPa, then decreased with further increasing the applied stress. On the other hand, *ε*_p_ increased in an exponential manner as the applied stress increased. As a result, the shape recovery ratio of the 50Ti alloy decreased continuously as shown in Figure 11c. For the 50Ti alloy, the shape recovery ratio of about 90% when the applied stress of 100 MPa decreased to 65% at 300 MPa, and then further decreased below 50% at 400 MPa. The 50.5Ti alloy exhibited very similar values of *ε*_r_ to those of the 50Ti alloy. Also, *ε*_p_ of the 50.5Ti alloy showed a similar response to the applied stress as that of the 50Ti alloy, but their magnitudes were slightly smaller than those of the 50Ti alloy, resulting in slightly larger recovery ratios. The 51Ti alloy exhibited much smaller values of *ε*_p_ and larger values of *ε*_r_ particularly at higher stresses, resulting in high recovery ratios and large work outputs when compared with the 50Ti and 50.5Ti alloys. 

Further Ti-rich side alloys, 51.5Ti and 52Ti, exhibited a different applied stress dependence of *ε*_r_ from the (50–51)Ti alloys. Specifically, *ε*_r_ of the 51.5Ti and 52Ti alloys continued to rise to a maximum value with increasing the applied stress. It is also seen that the 51.5Ti and 52Ti alloys exhibited excellent dimensional stability and shape memory properties—*ε*_p_ was almost zero and the shape recovery ratio was almost 100% at 300 MPa. Both alloys exhibited large values of *ε*_r_ with small values of *ε*_p_ even at 500 MPa, resulting in high work outputs. Similarly, in the Ti-lean alloys, dimensional stability was improved as the Ti content departed from the stoichiometric composition. In particular, the 49Ti alloy revealed superior dimensional stability and shape memory properties comparable with the 52Ti alloy. It is supposed that the improvement of dimensional stability is due to the formation of precipitates during thermal cycling. Consequently, it is concluded that the shape memory properties of the Ti–Ni–Pd alloys can be improved by the adjustment of the Ti content and precipitation hardening. 

## 4. Conclusions

In this study, the effect of the Ti content on the transformation temperatures, microstructures and shape memory properties during thermal cycling under constant stresses for Ti_x_Ni_70−x_Pd_30_ alloys with Ti contents in the range from 49 at.% to 52 at.% was investigated. The main conclusions are as follows:

(1) The Ti content of the matrix region increases linearly while the Ni content of the matrix region decreases with increasing the nominal Ti content. The martensitic transformation temperature shows the highest value when the Ti content in the matrix is near the stoichiometric composition and it decreases with increasing or decreasing the Ti content as compared to the stoichiometric composition.
(2) The martensitic transformation temperature decreases during thermal cycling and the degree of decrease in the transformation temperature becomes more pronounced as the composition of the alloy departs from the stoichiometric composition on both Ti-rich and Ti-lean sides. Ti_2_Pd and P phases are formed during thermal cycling in Ti-rich and Ti-lean alloys, respectively.
(3) Both Ti-rich and Ti-lean alloys exhibit excellent dimensional stabilities, higher recovery ratio and larger work output during thermal cycling under constant stresses when compared with the alloys with near-stoichiometric composition.
